# The Role of RhoJ in Endothelial Cell Biology and Tumor Pathology

**DOI:** 10.1155/2016/6386412

**Published:** 2016-07-31

**Authors:** Ting-Ting Shi, Gang Li, Hong-Tao Xiao

**Affiliations:** ^1^School of Medicine, University of Electronic Science and Technology of China, Chengdu 610054, China; ^2^Department of Pharmacy, Hospital of the University of Electronic Science and Technology of China and Sichuan Provincial People's Hospital, Chengdu 610072, China; ^3^Sichuan Translational Medicine Hospital, Chinese Academy of Sciences, Chengdu 610072, China

## Abstract

*Background*. RhoJ, an endothelially expressed member of Cdc42 (cell division cycle 42) subfamily of Rho GTPase, plays an important role in endocytic pathway, adipocyte differentiation, endothelial motility, tube formation, and focal adhesion. RhoJ is a selective and effective therapeutic target in tumor tissues or retinopathy.* Methods*. A systematic review was related to “small Rho GTPase” or “RhoJ” with “endothelial motility, tube formation and focal adhesion” and “tumor therapy”. This led to many cross-references involving RhoJ and these data have been incorporated into the following study.* Results*. We have grouped the role of RhoJ according to three main effects: RhoJ regulates endocytic pathway and adipocyte differentiation in early studies, and RhoJ shows an important role in endothelial cell biology; furthermore, RhoJ blockade serves as a target in tumor vasculature and enhances the effects of anticancer drug.* Conclusions*. More research is necessary to understand the role of RhoJ in many aspects, on the basis of current knowledge of the role of RhoJ blockade in tumor vessels, there are opportunities for the therapy of tumor, and RhoJ is expressed outside tumour vasculature and is involved in wound healing. Taking advantage of the opportunities could result in a development in tumor therapy.

## 1. Introduction

Like the majority of Ras superfamily proteins, Rho family small GTPases are molecular switches that cycle between an active GTP-bound and inactive GDP-bound form [1]. In the GTP-bound state, these Rho proteins bind to a large collection of downstream effector molecules, thereby simulating a variety of signaling cascades that promote general cellular responses such as morphogenesis, cytoskeletal change, migration, microtubule dynamics, vesicle trafficking, cell polarity, division and adhesion, and cell cycle progression [1–3].

RhoJ, also known as TCL (TC10-like), was first identified 16 years ago, which shared 85% and 78% amino acid similarity to TC10 and Cdc42, respectively [4]. Shortly afterwards, TC10*β* was reported with 70% sequence identity to TC10 of mouse, and another variant termed TC10*β*Long (TC10*β*L) [5], an isoform of TC10*β* [6], was discovered. Subsequently Nishizuka et al. [6] isolated the cDNA of mouse clone 26 that is identical to mouse TC10*β*L and also the same length as human TCL, so they refer to clone 26 as TCL/TC10*β*L. Further report notes that RhoJ belongs to the Cdc42 subfamily of Rho GTPases [1]. Like TC10 and Cdc42, RhoJ binds the CRIB (Cdc42/Rac interactive bind) domains of WASP and PAK [4].

The potential function of RhoJ was initially interrogated by early studies that suggested that RhoJ played a role in modulating the formation of distinct cytoskeletal structures [4] and lamellipodia [7] as well as in actin filaments [8]. A role for RhoJ in the early stage of adipocyte differentiation was also identified; the constitutive overexpression of RhoJ was found to be sufficient to cause the differentiation in vitro, whereas knockdown of RhoJ inhibited this process [6]. Also, RhoJ has been shown to regulate early endocytic pathway [9].

Subsequently it was identified as being highly and specially expressed by endothelial cells [10–13] and was regulated by the EST family transcription factor ERG [13]; functionally, RhoJ has been reported to modulate endothelial motility, tube formation in vitro [12], and vascular morphogenesis in vivo [13]. In addition, RhoJ is also important for vascularisation [11, 14, 15], especially for tumor angiogenesis [16, 17]; RhoJ is the common downstream target of the Sema3E signals [11] in regulating retinal vascularisation; the knockdown of RhoJ causes a double assault on tumor vessels by both the inhibition of angiogenesis and vascular disruption [16]. The molecular mechanism showed that RhoJ localized to focal adhesions, modulated their numbers, and negatively regulated actomyosin contractility and stress fibers formation [12]. Further study suggested that RhoJ interacted with the GIT-PIX complex and modulated focal adhesion disassembly [17].

Recently, the role of RhoJ has been explored in regulating the cell migration and invasion of melanoma by altering actin cytoskeletal dynamics [18], which is consistent with observations that RhoJ modulates actomyosin contractility [4, 12]; the same group suggested RhoJ as a linchpin determinant of melanoma that regulates chemoresistance by activating PAK1 [19].

The aim of this review was to characterize the cellular and physiological functions of RhoJ and molecular mechanism by which RhoJ regulates adipocyte differentiation, endothelial motility, tube formation, and focal adhesion turnover. Additionally, we further shed light on the fact that RhoJ was a selective and effective therapeutic target in tumor tissues or retinopathy.

## 2. Methods and Materials

Our bibliographic sources were the various SciFinder updated to 2016 June. In order to assess the data in detail, we used the search terms “RhoJ” with “endothelial motility, tube formation and focal adhesion” and “tumor therapy”. Papers were restricted to those published in the English languages and “paper” and “review” as the document type.

## 3. Results and Conclusions

### 3.1. RhoJ Regulates Adipogenesis

The role of RhoJ in the early stage of adipocyte differentiation was initially demonstrated 13 years ago, suggesting that it probably linked to the peroxisome proliferator-activated receptor *γ* (PPAR*γ*) [6]; however, the relationship between RhoJ activity and the early stage of this differentiation was also unclear.

Subsequently, Kawaji et al. [20] have identified an inhibition of mitotic clonal expansion (MCE) by RhoJ knockdown in adipogenesis. In addition, the suppression of RhoJ repressed the incorporation of bromodeoxyuridine (BrdU), indicating that DNA synthesis was prevented by the suppression; furthermore, the knockdown of RhoJ inhibited the expression of the C/EBP *β* and C/EBP *δ* (CCAAT/enhancer-binding protein) during MCE [20]. A-C/EBP, which forms stable inactive heterodimers with C/EBP*β*, has been reported to prevent the downregulation of p27/Kip1 (cyclin-dependent kinase inhibitor) expression and impaired MCE [21]; further report showed the potential role of RhoJ regulating MCE via cyclinD1, indicating the important role of RhoJ in controlling p27/Kip1's degradation for MCE [20]. Thus, the knockdown RhoJ may influence cell cycle.

### 3.2. The Role of RhoJ in Endothelial Cell Biology

#### 3.2.1. The Function of RhoJ in Endothelial Cells Biology and Angiogenesis

RhoJ has a vascular expression pattern that is distinct from other Rho GTPases [12, 13]. It is highly enriched in all endothelial cells, including venous, arterial, and microvascular ECs [13]. RhoJ also was expressed in some nonendothelial cells, including liver, muscle, and some cancer cells, indicating that RhoJ expression was not endothelium-specific [12], whereas these levels were much lower than in ECs.

The expression patterns of human, zebrafish, and mouse have exhibited species differences in the regulation of RhoJ expression. For example, whole-mount in situ hybridization performed a vascular expression pattern of RhoJ in the developing mouse at embryo day 9.5, a stage at which the angiogenesis is occurring and vasculature is developing, and RhoJ was expressed in the intersomitic vessels and main trunk vessels [12]. However, another study reported that the single zebrafish orthologue of RhoJ did not possess a vascular expression pattern and instead was transiently expressed in the somites at 24 hpf (hours after fertilization) and in the cephalic region at 48 hpf [22]. Additional instance, RhoJ was found to be expressed in B-cells and mouse platelets, but not in numerous human B-cell lines or platelets [22].

Initially, studies in Hela cells demonstrated that RhoJ localized to the plasma membrane and the early/sorting endosomes (EE or ES) [9], suggesting a role in the early endocytic pathway. Knockdown of RhoJ in Hela cells using small interfering RNA (siRNA) did not affect receptor-dependent internalization of transferrin (Tf), whereas Tf accumulated in Rab5-positive uncoated endocytic vesicles and failed to reach the EE antigen-1-positive early endosomal compartments and the pericentriolar recycling endosomes [9]. Recently, using anti-vinculin antibodies or anti-phospho FAK antibodies, indicating RhoJ was localized in punctuate regions of cell, these structures were focal adhesions [12]; furthermore, daRhoJ (dominant-active RhoJ) primarily localized to the plasma membrane, where it was more concentrated in focal adhesions; there was also some localization to intracellular vesicles. Subsequently, it also has been observed that transfected or virally transduced wild-type RhoJ and daRhoJ had a vesicular location pattern, especially when expression levels are very high [22]. Thus, RhoJ possibly drove its localization to intracellular vesicles due to the fact that focal adhesions were internalized by endocytosis [23].

The functions of RhoJ in endothelial cells were also reported. Using HUVECs with either overexpression of dominant-active RhoJ or siRNA-mediated knockdown of RhoJ has demonstrated that this Rho GTPase played a role in endothelial cells biology and angiogenesis [12]. During angiogenesis, ECs perform a variety of functions, including degradation of the extracellular matrix, motility, proliferation, lumen formation, and vessel stabilization [24]. Knocking down RhoJ resulted in impaired cell motility and diminished proliferation, whereas daRhoJ promoted cell migration [12]; moreover, RhoJ knockdown resulted in highly impaired tube formation, the tubes were significantly shorter, fewer, and lesser branched. This inhibition in tube formation is consistent with those of Yuan et al. [13], who found that knockdown of RhoJ resulted in a marked reduction in the ability of ECs to form lumens; in addition, they demonstrated that RhoJ was a target of ERG; knockdown of ERG expression in ECs led to a reduction in the expression of RhoJ. Recently, Richards et al. [25] demonstrated that RhoJ and FMNL3 were required for polarized trafficking of podocalyxin to the early apical surface, indicating it was important event in vascular lumenogenesis. Furthermore, knockdown of RhoJ did not influence the expression of the other Rho GTPases, RhoA, Rac1, or Cdc42, indicating that RhoJ has a distinct role in ECs [12].

Then, the related downstream signal of RhoJ was elaborated in ECs angiogenesis. Yuan et al. [13] demonstrated that RhoJ could coprecipitate with Cdc42, suggesting that they may interact directly or with a common binding partner. In another study, it appears that they interact in a multicomponent complex that may be critical for proper EC lumen formation [26]. Furthermore, Yuan et al. [13] observed that suppression of RhoJ leads to reciprocal effects on Rho GTPases activation with marked induction of RhoA activity and suppression of Rac1 activation. Rac1 are required for lumen formation, whereas RhoA is not [27, 28]. During EC tube formation, knockdown of RhoJ significantly decreased the phosphorylation of protein-activated kinase Pak2 and Pak4 [13] that were activated by Cdc42. Recently, it has been demonstrated that FMNL3 acted downstream of RhoJ to regulate polarized trafficking of podocalyxin to the endothelial AMIS (apical membrane insertion site) [25].

Similarly, the related upstream signal of Cdc42 and RhoJ was demonstrated in ECs angiogenesis. During angiogenesis, ECs coordinates lumen formation [24] and tube formation is VEGF- (vascular endothelial growth factor-) dependent in the coculture assay [29], and VEGF is one of the most potent proangiogenic factors. Therefore, the connection between Cdc42, RhoJ, and VEGF was elaborated separately.

In ECs, Cdc42 is activated by binding of VEGF to VEFGR2 indirectly [30]; this finding is consistent with those of Kaur et al. [12], who found that VEGF induced activation of Cdc42 peaked at 15 min (minutes) by pull-down of the active GTP-bound forms of the PAK1 CRIB domain versus cellular lysates, whereas binding of semaphorin 3E (Sema3E) to PlexinD1 receptor inactivates Cdc42 [11].

Furthermore, the activation of Rho GTPases can be regulated through activity of a number of cell-surface receptors including integrins, receptor tyrosine kinases, and G-protein-coupled receptors. These in turn influence activity of GAPs (GTPase-activating proteins) and GEFs (guanine-nucleotide-exchange factors); GEFs are activatory regulators which mediate the exchange of GDP to GTP, whereas GAPs promote the intrinsic GTP hydrolysis of Rho GTPases; therefore GEFs can activate the Rho GTPases. Arhgef15 (also known as Vsm-RhoGEF [31] or Ephexin5 [32]), an EC-specific GEF, promotes retinal angiogenesis by mediating VEGF-induced Cdc42 activation [15]; the results display that VEGF-induced activation of Cdc42 was abrogated by Arhgef15 knockdown in HUVECS (human umbilical vascular ECs).

By contrast, RhoJ is inactivated by VEGF and activated by Sema3E-PlexinD1 in ECs [11]; it was found that VEGF reduced the level of Rho-GTP after the earlier time of 2 min by higher concentration (50 ng/mL) of VEGF; Kaur et al. [12] found that 10 ng/mL VEGF induced a very slight reduction in RhoJ-GTP levels after 1 min with no significant difference compared to the 0 min; however, they assessed 10 ng/mL VEGF activate RhoJ at 15–30 min: the levels of activated RhoJ were 2-fold increased, a more modest, slower, and more sustained activation. This suggests that VEGF of high dose may inactivate RhoJ at early time, whereas VEGF activate RhoJ at a later time with a durable activation; therefore, VEGF may activate RhoJ in general. Additionally, a study has demonstrated that Arhgef15 potentiated RhoJ inactivation in 293T cells but not ECs [15]; thus, the role of Arhgef15 in regulation of RhoJ is still be further explored.

**Figure 1:**
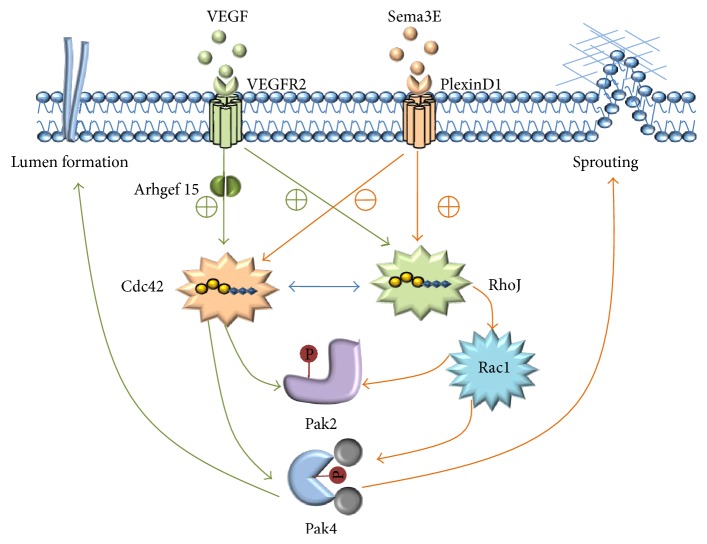
The inverse regulation of the activation status of RhoJ and Cdc42 is modulated via VEGF-VEGFR2 and Sema3E-PlexinD1 signals. Cdc42 is activated by Arhgef 15, which stimulates Pak2 and Pak4 phosphorylation and lumen formation. RhoJ leads to suppression of Rac1 activation, which is associated with the sprouting of cell.

Thus, the regulation of the activation status of RhoJ and Cdc42 downstream of VEGF-VEGFR2 and Sema3E-PlexinD1 signals are the pivotal intracellular events to angiogenesis in ECs [13]. Thus, a model for the function of RhoJ in endothelial cells biology and angiogenesis was depicted in Figure 1.

#### 3.2.2. RhoJ Modulates the Stress Fibers and Focal Adhesions

Previous study demonstrated that increased actomyosin contractility resulted in cell death and cell detachment in the tissue culture organotypic angiogenesis assay of tubule formation [33]. Thus, the role of RhoJ in stress fibers and focal adhesions was further described. Stress fibers are contractile actomyosin structures, and actomyosin describes the complex of actin filaments; therefore, stress fibers are bundles of numerous actin filaments [34]. The actin cytoskeleton modulates cellular shape; this regulation is crucial to cellular movement which requires both protrusion and contractility of cell membrane. Protrusions of cell membrane are stabilized by focal adhesions that combine with modulation of the actin cytoskeleton. Focal adhesions are large, dynamic protein complexes that mediate connections between the intracellular cytoskeleton and extracellular matrix. Integrin-mediated adhesion regulates cell motility, survival, and cell cycle progression [35]. There is a reciprocal regulation and complex interplay between focal adhesion maturation, turnover, and actomyosin contractility; thus, adhesion assembly and disassembly drive the migration cycle by activating Rho GTPases, which in turn regulate actin contractility and myosin II activity, and, conversely, regulating myosin II activity can influence focal adhesion distribution and size [36]; thus, RhoJ may regulate contractility via dynamic regulation of focal adhesion assembly and turnover. Therefore, the molecular mechanism of RhoJ was demonstrated separately in stress fibers and focal adhesions.

A number of studies have suggested that mutant forms of RhoJ can regulate the formation of cytoskeletal structures in a range of cell types. Fibroblastic cells expressing daRhoJ were shown to display strong and localized F-actin accumulation, as well as a reduction in actin stress fibers [4]. In PAE (porcine aortic endothelial) cells, daRhoJ triggered the formation of lamellipodia and bundles of actin filaments; in addition, the cells expressing daRhoJ had a number of dotted filamentous actin-containing structures in the lamellae [7]. In another study, also in PAE cells, daRhoJ induced the formation of podosome that had punctuate actin-rich adhesion structures [37]. Subsequently, Monypenny et al. [38] demonstrated that RhoJ knockdown reduced migration speed, but RhoJ, Tc10, Wrch1, Rac2, and Rac3 were not required for the directional response toward PDGF- (Platelet-Derived Growth Factor-) dependent chemotaxis in primary fibroblasts; on the other hand, Hou et al. [39] demonstrated that inhibition of RhoJ (TCL) interfered with the cell migration and polarity in human corneal epithelial large T antigen cells (HCET). Furthermore, it has been demonstrated that daRhoJ resulted in the reduction of contractility, knockdown RhoJ had the opposite effect, and expression of knocking down RhoJ was found to increase the number of stress fibers in HUVECs (human umbilical vein endothelial cells) at the wound edge, whereas daRhoJ expression reduced the levels of stress fibers in HUVECs at the migration front; however, no differences were observed in HUVECs in the monolayer [12]. The increased stress fiber formation may reflect increased actomyosin contractility. It has been shown that quiescent tubes have an increased contractility, whereas the reduction of contractility was required for sprouting tubes [40]. Thus, knockdown of RhoJ increases stress fibers and then inhibits tubular sprout and motility.

The molecular mechanisms of RhoJ in stress fibers were investigated. The small Rho GTPase, RhoA, RhoB, and RhoC, when activated, regulate stress fiber formation via their activation of ROCK (Rho-kinase) I/II (or ROCK *β*/*α*); ROCK phosphorylates a number of targets, resulting in increased MLC (myosin light chain) phosphorylation and increased actomyosin contractility [41]. It has been shown that RhoA-ROCK-myosin signaling is an important regulator of stress fiber formation and EC contraction [42]. Elevated level of phosphorylated MLC2 was found in established tubules and sprouting tubules, indicative of higher Rho-kinase activity [33]. Furthermore, Abraham et al. [40] showed that VE-cadherin antagonized VEGFR2 signaling, and consequently, inhibition of VE-cadherin, Rho-kinase, or actomyosin contractility leads to VEGF-driven, Rac1-dependent sprouting [40], suggesting that this early stage of the sprout requires decreased actomyosin contractility, whereas the stabilization of newly established vessels requires increased contractility. These findings are consistent with early study, which found that ERK-MAPK promoted endothelial cell sprouting and survival by downregulating Rho-kinase signaling [33]. Another crucial member of Rho GTPase, RhoJ, was also investigated; RhoJ knockdown results in an increase in MLC phosphorylation; additionally, using 2 structurally unrelated inhibitors of nonmuscle myosin II or inhibitors of ROCK, the motility defect of RhoJ siRNA-treated endothelial cells was reversed in both the Matrigel tube forming and scratch-wound assays [12].

RhoJ activity also modulates focal adhesion numbers. It has been demonstrated that RhoJ activity promoted focal adhesion disassembly [17] and is consistent with previous studies that RhoJ modulates focal adhesion numbers [12, 22]: knockdown of RhoJ induces significantly more numerous and smaller more stable focal adhesion owing to slower disassembly in cells migrating at the edge of scratch, but not in cells within a monolayer, and daRhoJ causes decreased numbers and large size of adhesions owing to more rapid disassembly.

Recently, the molecular mechanism by which RhoJ regulates focal adhesion dynamics was found [17], which demonstrated that the active form of RhoJ interacted with the SHD (Spa homology domains) of GIT1 (G-protein-coupled receptor kinase-interacting target) proteins to promote the recruitment of the GIT-PIX (Pak-interacting exchange factor) complex to focal adhesion. This finding is consistent with those of Kuo et al. [43], who found that *β*-PIX derived nascent focal adhesion turnover and siRNA knockdown of *β*-PIX decreased focal adhesion disassembly. Furthermore, the early study suggested that GIT proteins are recruited to focal adhesions through their binding of paxillin [44], and GIT proteins associated with PIX proteins coupled to FAK (focal adhesion kinase) through SHD, which in turn resulted in the recruitment of the kinase PAK to focal adhesions; the latter required the activation of PAK1, and it will be of interest to discover whether the GIT-PIX complex is also involved.

### 3.3. RhoJ: The Target in Tumor Vasculature

#### 3.3.1. The Role of RhoJ in Tumor Angiogenesis and Various Solid Tumor Models

The members of Rho family of small GTPases have been discovered as key regulator of angiogenesis, regulating a diversity of cellular processes and homeostasis [45]. Rho GTPases are essential downstream target for VEGF-mediated angiogenesis in endothelial cells and also are involved in tumor cell invasion; moreover, a well-controlled balance between different Rho GTPases governs almost all aspects of angiogenic processes such as ECs migration, proliferation, vascular permeability, extracellular matrix remodeling, morphogenesis, and survival [45, 46]. Thus, novel therapeutic strategies are discovered that the interference of Rho GTPase signaling serves as a target for anticancer therapy via interference with the angiogenesis and invasion of tumor.

RhoJ is a small Rho GTPase mainly expressed in ECs [12, 13]. During development, RhoJ is specifically expressed in the intersomitic vessels and dorsal aorta of mouse embryos as well as in the retinal vessels of the postnatal mouse [11, 12]. Neuron-derived Sema3E signaled to PlexinD1 and activated RhoJ, thereby selectively suppressing disoriented outgrowth of extraretinal vessels and leading to the subsequent regeneration of normal vasculature in ischemic retinas [11]. Also, RhoJ knockout has been found to affect neonatal retinal vascularisation [14].

And recent study has revealed that high RhoJ was expressed in tumor ECs, whereas some non-ECs also occasionally expressed RhoJ, such as tumor stromal cells and perivascular mural cells [16]. The rapid growth of tumors is crucially dependent on the development of vessels to support the tumor cell proliferation [47]; another study suggested that RhoJ is required to facilitate this process [17]: knockout of RhoJ has been found to result in reduced vessel density and diminished tumor growth, which is consistent with its role in endothelial tube formation in vitro. Additionally, RhoJ has been identified as part of the common tumor angiogenesis signature, one of top 20 genes strongly upregulated in tumor vessels [48]. These findings were consistent with those of Kim et al. [16], who found that the tumor blood vessels in the granulation area of wound displayed high RhoJ expression, and RhoJ-KO mice showed delayed wound closure, reduced vascular density, and decreased granulation area in the wound regions compared with RhoJ-WT mice, suggesting that RhoJ deficiency delayed wound healing through attenuated angiogenesis. Moreover, in tumor vasculature, the expression of RhoJ follows a distinct spatiotemporal regulation, and it is most robustly expressed during early tumor progression, by contrast, being attenuated in later stages of tumor growth [16]. Furthermore, targeted RhoJ deletion suppressed tumor angiogenesis and disrupted tumor vessel integrity in tumor ECs [16]; it was shown that the impact of EC-specific RhoJ deletion on tumor growth was less compared to global RhoJ deletion; additionally, compared to RhoJ-WT^EC^, LLC tumors of RhoJ-KO^EC^ showed reduced growth and remarkable intratumoral hemorrhagic necrosis; vascular densities of RhoJ-KO^EC^ tumors were reduced in the peri- and intratumoral areas. Thus, in tumor ECs, RhoJ is critical to the maintenance of tumor vascular integrity and regulation of tumor angiogenesis.

The role of RhoJ has been demonstrated in various solid tumor models. Recently, in melanoma cell lines, it was suggested that not only did RhoJ regulate chemoresistance by suppressing the pathways of DNA damage sensing [19] but also it modulated their motility and invasion by altering actin cytoskeletal dynamics [18]. Compared to primary melanomas, RhoJ is overexpressed in advanced melanomas, and RhoJ activates PAK1 in response to drug-induced DNA damage; the activation of RhoJ/PAK1 allows melanoma cells to tolerate higher levels of DNA damage; thus, tumor cells have a profound resistance to DNA damage agents [19]. Additionally, RhoJ depletion inhibited melanoma tumor growth and lymphatic spread in vivo and melanoma cell migration and invasion in vitro [18]; this finding is consistent with the established roles of Rho GTPase in melanoma cell motility and invasion [49, 50]. Similarly, RhoJ modulates melanoma cell migration by altering actin cytoskeletal dynamics via inducing the phosphorylation of cofilin, LIMK, and p41-ARC (ARP2/3 complex subunit) in a PAK1-dependent manner in vitro. Taken together, the inhibition of RhoJ would be a strategy that could both suppress melanoma metastasis and invasion and also sensitize tumors to DNA damage agents.

In the LLC (Lewis lung carcinoma) tumors, RhoJ deletion inhibits tumors growth, neovessel formation, and metastasis [16]. Using LLC tumor model by injecting LLC cells into RhoJ-KO and RhoJ-WT mice, RhoJ-KO mice displayed a most prominent reduction during early tumor growth, and the tumor had an increased hemorrhagic foci, especially in the intratumoral area; furthermore, hypoxia was more evident with extensive apoptosis in the central tumor and vascular densities were less in the peri- and intratumoral areas; most importantly, the tumor vascular sprouting was lower, suggesting that RhoJ is important for neovessel formation by promoting sprouting angiogenesis; finally, it has been shown that the number of metastatic tumor colonies in the lung was less and has showed less metastasized LLC tumor cells in the LNs (lymph nodes) of RhoJ-KO mice [16].

Similarly, in the spontaneous breast cancer model, RhoJ deletion also reduces tumor growth, neovessel formation, and metastasis [16]. Compared to RhoJ-WT, RhoJ-KO showed reduced development of spontaneous tumor nodules, the number of nodules per mouse decreased, and median time to palpable tumor development was delayed; furthermore, the tumor burden and average size were less than control, and histological examination has shown that RhoJ blockade resulted in more noninvasive carcinoma lesions with well-preserved tumor margins, indicating that RhoJ deletion delays tumor invasion and progression; also, the tumor vascular sprouting was less and vascular densities were less in the peri- and intratumoral areas; moreover, morphology of tumor vessels in the intratumoral regions seemed more disrupted and tumor vasculatures were highly permeable; finally, the number of metastatic tumor colonies in the lung was less [16].

Taken together, RhoJ plays a crucial role in the maintenance of tumor vascular integrity and formation of tumor neovessels, affecting the tumor progression.

#### 3.3.2. RhoJ Blockade Enhances the Effects of Anticancer Drug

RhoJ expression in normal tissues of adult mice was very distinct and infrequent, only occasionally appearing in stromal cells and heart blood vessels and in LN blood vessels, and RhoJ-KO mice grew to adulthood normally without any vascular abnormalities or growth retardation in major organs such as heart, lung, kidney, and liver [16]. However, RhoJ was also expressed in ECs lining vessels in a number of adult human tissues (heart, lung, muscle, liver, lymph node, placenta, pancreas, bone cancer, bladder cancer, and ovarian cancer) [12], especially in heart and lung [13], and mouse embryos, but it was absent from tissues and tumors of testis, colon, brain, stomach, kidney, or rectal cancer [12]. Furthermore, in human tissues, RhoJ expression in normal colon tissues is undetectable, but it is highly expressed in the tumor vessels of colon adenocarcinomas; moreover, it has been found that the patients that had colon cancer with high RhoJ expression had decreased overall survival and increased prevalence of lymphovascular invasion [16]. These findings indicate that RhoJ may be a promising candidate for a more selective vascular targeting therapy compared to current chemotherapies.

As is previously known, anticancer drug resistance limited the ability of drug to penetrate the intratumoral core of tumors and to reach overall tumor cells in a potentially lethal concentration [51], because abnormal organization and the structure of tumor vasculature and elevated tumor interstitial fluid pressure limited anticancer drug to deliver to this core [52]. In addition, heterogeneity within the tumor microenvironment results in marked gradients in the rate of cell proliferation and areas of acidity and hypoxia, all of which affect the sensitivity of tumor cells to drug chemotherapy [51]. However, RhoJ blockade preferentially induces vascular shutdown of intratumoral regions and results in necrosis of the tumor cells [16]. By combining cisplatin and the RhoJ blockade, cisplatin profoundly retards tumor growth in RhoJ-KO mice; the combination was proved to be effective in delaying tumor progression, indicating that RhoJ deletion in combination with conventional chemotherapeutic drugs could enhance antitumor effect [16]. Therefore, the effect of RhoJ deficiency was further explored in concert with conventional anticancer drugs.

Vascular targeting agents are commonly classified as either VDAs (vascular disrupting agents) or AIAs (angiogenesis-inhibiting agents) during past decade. The adjuvant of RhoJ blockade was demonstrated separately in VDAs and AIAs.

VDAs are known to disrupt preformed tumor vessels by directly affecting the cytoskeletons of ECs [53] and shut down blood flow, subsequently resulting in massive tumor hemorrhage and necrosis [54]. VDAs are particularly effective in the intratumoral regions of established tumor vasculature either by direct apoptotic effects or by the influence of tubulin cytoskeleton [53]; moreover, VDAs induce activation of RhoA ROCK signaling in tumor ECs. However, despite promising preclinical results, they failed to display efficacy in clinical trials [55]. Furthermore, the main drawback of VDAs is that they fail to target the remaining peripheral viable rim and even resist VDAs [54]. Instead, RhoJ blockade increased shutdown of preexisting tumor vasculatures in the intratumoral areas and exerted its antitumor effect in both the peri- and intratumoral regions [16]; also, RhoJ deletion shares a common mechanism with VDAs, positively regulating the RhoA-ROCK signaling pathway. Indeed, it has been confirmed that RhoJ blockade may overcome the limitation of current VDA monotherapies, such as CA4P [16]; in RhoJ-KO mice, treatment with CA4P showed an additional inhibition in tumor growth and reduced vascular densities, and the tumor metastatic effect was significantly less. Therefore, RhoJ blockade is a valuable complementary therapy to overcome the current resistance acquired from VDAs.

AIAs mainly induce tumor vasculature normalization and suppress the formation of tumor neovessels [54]. AIAs are most effective in the peritumoral regions of newly progressing tumors where new tumor vessels are robustly developing, and they selectively reduce immature vessel numbers [53], whereas current AIAs affect normal vessels as well, because their primary targets, VEGF-A and its downstream effectors molecules, are expressed ubiquitously. Thus, they induce systemic adverse effects including hemorrhage, the disturbance of homeostasis in the cardiovascular and renal systems, and tissue repair and wound healing [56, 57]. However, RhoJ expression is very specific to pathologic conditions, especially in tumor tissues, while being rarely expressed in major organs under normal physiologic conditions; moreover, the combination therapy of RhoJ blockade and VEGF decoy receptor, VEGF-trap (AIA therapies), displayed comparatively potent antiangiogenic effect in both peri- and intratumoral regions of the LLC tumor [16]: compared to monotherapy, the combination increased the effect in decreasing tumor volume, significantly decreased tumor vessel densities, and showed a dramatic reduction in LN metastasis. On the other hand, tumor vasculatures regrow alongside the ghost tracks of residual BM (basement membrane) after cessation or during the dormant period of AIA treatment [58]. However, it has been observed that RhoJ-deficient tumor vessels showed a severe loss of BM, suggesting that concurrent RhoJ deficiency could abolish residual BM in concert with AIAs and impede the regrowth of tumor vessels, consequently, leading to a sustained response to AIA therapies [16].

Together, RhoJ blockade encompasses the aspects of both AIAs and VDAs and provides an effective strategy for targeting tumor vessels. The “double assault” of RhoJ blockade on tumor vessels showed that it simultaneously suppresses the formation of tumor neovessel and disrupts the preperformed tumor vessel work [16]. Thus, RhoJ blockade compensates for and augments anticancer drug therapies.

## 4. Future Perspectives

A number of outstanding questions about the function of RhoJ remain. Kim et al. [16] indicated that RhoJ played a positive angiogenic role during wound healing, which may be an unavoidable side effect of a putative RhoJ inhibitor; moreover, they used the APT_EDB_-liposome (a aptide specific for extra domain B was conjugated with liposome) complex as a carrier, and they effectively delivered siRhoJ into tumor tissues and dramatically delayed tumor growth and metastasis, especially in concert with VEGF-trap; therefore, they established a way to clinically inhibit RhoJ. However, further development of specific RhoJ inhibitors is still needed to ascertain their safety and efficacy in clinical settings.
